# A Comparison of 3D and 2D FE Frictional Heating Models for Long and Variable Applications of Railway Tread Brake

**DOI:** 10.3390/ma13214846

**Published:** 2020-10-29

**Authors:** Michal Kuciej, Piotr Grzes, Piotr Wasilewski

**Affiliations:** 1 Faculty of Mechanical Engineering, Bialystok University of Technology (BUT), 45C Wiejska Street, 15-351 Bialystok, Poland; p.grzes@pb.edu.pl; 2 Frimatrail Frenoplast S.A., 15 Watykańska Street, 05-200 Majdan, Poland; pwasilewski@frimatrail-frenoplast.pl

**Keywords:** temperature, railway tread brake, 1xBg brake configuration, long-term braking, finite element method

## Abstract

The article proposes two 3D and 2D numerical FE models of frictional heating for the estimation of temperature distributions in railway tread brake in 1xBg configuration during repeated long-term braking. The results of computations were compared with the time courses of temperature measured using thermocouples throughout the duration of the tests on a full-scale dynamometer for two different brake shoe materials in combination with a steel wheel. The resulting temperature distributions calculated using the proposed models agreed well with the experimental measurements, and the maximum difference in temperature values does not exceed 20%. It has been proven that 2D FE model can be as efficient as 3D model to estimate the temperature distribution during long-term and variable braking in the considered friction node. The differences in the calculation of the temperature values using these models did not exceed 3%, and the calculation time for the 2D model, compared to the 3D model, was shorter approximately 85 times for the braking cycle lasting 5032 s, and approximately 45 times for the braking cycle lasting 3297 s.

## 1. Introduction

Despite the development of railway technology, tread brake, designed over 100 years ago, is still a widely used solution in railway vehicles, in particular in freight wagons and vehicles where available space is a significant limitation in the structure, e.g., in motor bogies of motive power units or in subway vehicles. One of the most important advantages of the tread brake is its simple design and high reliability. In a typical pneumatic railway brake, the force exerted on friction elements is generated by compressed air in the brake cylinder. In tread brake, the force from the brake cylinder is multiplied by brake rigging and presses the brake block (equipped with brake shoe) against the wheel running surface. The heat generated leads to rapid increase in temperature on the friction surfaces of brake components and other parts of the brake assembly.

The condition of tread-braked wheel running surface is significantly influenced by the material, from which the brake shoe is made. One of the important factors that led to the development of composite brake blocks in Europe was an attempt to reduce rolling noise of wheelsets, which is closely related to roughness of their running surface [1,2]. The use of composite brake shoes reduces the roughness of wheel running surface [2,3], and experimental studies have shown that in comparison to cast-iron, composite brake shoes also have less tendency to generate so-called hot spots [1]. Additionally, because of lower thermal conductivity, composite brake shoes increase the amount of heat absorbed by the wheel [4].

Manufacturers of railway braking systems verify friction pair selection on full-scale inertia dynamometer, i.e., such on which the friction pair is tested in its normal size. During the tests of this type, the actual operating conditions of the brake are simulated, including the routes of the proposed railway vehicles. The determined friction pair characteristics are evaluated, e.g., what is the relationship between the value of the coefficient of friction or wear and temperature, sliding velocity, pressure, or braked mass. The condition of the friction elements is also analyzed, i.e., whether signs of thermal degradation of the material or its damage are observed under given operating conditions. Dynamometer tests are also a stage of the process to receive approval granted by UIC (Union Internationale des Chemins de fer—International Union of Railways) to friction elements [5], and are also performed in the course of conformity assessment required by European law, among others, for brake shoes used in freight wagons [6,7].

Full-scale railway brake dynamometers are also used to determine temperature of friction elements in the course of brake application. In the article [8], Kim studied possible applications of infrared thermography in the characterization of railway components. It was successfully used to measure temperature of railway brake disc surface as well as to detect hot spots. It has been shown that the infrared technique allows for high-resolution measurement of the temperature field measured in real time and can complement the measurements with sliding thermocouples, the disadvantage of which is inaccurate measurement due to friction and the occurring heat generation. Paper by Hong et al. [9] is focused on investigating the mechanism of hot bands and hot spots formation in railway brake disc during high-energy brake applications. Experimental measurements, performed on a full-scale dynamometer, were employed to study so-called braking patterns which are observed throughout repetitive braking process. An attempt to simulate hot band formation in an FEM (finite element method) model has also been made by the authors and lead to the conclusion that taking into account one-sided wear of the pad would improve the agreement between the experiment and FE analysis.

Additionally, reduced-scale dynamometers are used to study tribological phenomena related to railway braking. In the article [10] Tang et al. modified contact interface of the friction block to analyze its influence on temperature fields formed during braking on the counter-face element as well as wear of the friction block and tendency to generate squeal. Rokhim et al. employed reduced-scale dynamometer to characterize frictional properties of composite railway brake shoe, namely dependence of the coefficient of friction on sliding velocity and contact pressure [11]. It has to be noted, however, that simulation of the operating conditions of the railway vehicle in reduced scale requires very careful design of the experiment, including geometry of the friction pair [12].

Due to the cost of conducting tests on dynamometer stands, and bearing in mind how important it is to determine the operating temperature and thermal loads of the friction pair for given parameters of railway vehicle operation, the object of interest of both scientific research and industry is modeling of the frictional heating process. This is evidenced by the multitude of papers published so far, the subject of which are experimental studies of temperature fields as well as analytical and numerical modeling of the frictional heating process.

A two-dimensional axisymmetric FE model to determine the mean temperature on the surfaces of the brake shoe and wheel was proposed in the papers [4,13]. The partition of heat flux between friction pair elements depended on the thermal resistance. The major advantage of the model is that it accounts for wheel-to-rail heat flow.

The model presented by Vernersson in the article [13] was calibrated by Teimourimanesh et al. using the results of temperature measurement obtained during the dynamometer test and passage of a metro vehicle [14]. Teimourimanesh used it afterwards to perform calculations of the friction pair temperature evolution for a metro vehicle [15]. The material of the wheel (ER7 steel) was thermosensitive, i.e., thermophysical properties depended on temperature. A constant and equal deceleration value was established for all braking cases, and—as a consequence—a constant value of friction generated at the wheel-brake shoe interface. The results of calculations of temperature changes were the basis for calculations of thermomechanical loads on the wheel. The analysis of the wheel mechanical loads was used to estimate the wheel designed service life (predicted mileage requiring replacement).

Petersson developed a two-dimensional model of the railway tread brake with a single brake shoe using FEM [16]. The model was calibrated on the basis of temperature measurements made during the test on a full-scale dynamometer. Using the model, the author examined the effect of heat flux distribution between the wheel and the brake shoe, pressure distribution and heat capacity of the brake shoe on the calculated temperature fields. Increasing the value of brake shoe heat capacity set in the model did not significantly affect the calculated value of the wheel temperature, but had a significant impact on the value of the brake shoe temperature

In the paper [17] Milošević et al. used FEM to develop a three-dimensional model of a frictionally heated wheel with a tread brake in 2xBgu configuration (bilateral configuration, two brake shoes per side of the wheel). The temperature field changes were calculated numerically for six variants of continuous brake applications with different values of maintained velocity and pressure exerted on the brake shoes. It was assumed that the coefficient of friction of the wheel-brake shoe system is constant during all brake applications and independent of the velocity and pressure. In the developed brake numerical model, perfect thermal contact between the wheel and the brake shoes was established (equal temperature on the contact surfaces).

In the article [18] Suchánek et al. employed FEM to calculate temperature of railway wheel in the course of two consecutive applications of a tread brake. The calculated temperature values were subsequently used to determine equivalent stress in the wheel. One of the major simplifications of the FE model presented in this study was that rotational motion of the wheel was not accounted for.

Handa et al. combined full-scale dynamometer test and FE model to investigate hollow wear of wheel running surface characteristic of tread-braked railway wheels [19]. It was concluded that the main factor contributing to this specific wear pattern is plastic deformation caused by rolling contact on the part of the wheel profile which is heated during brake application.

Additionally, disc brakes are the subject of scientific research concerning mathematical modeling of frictional heating. A few papers are shortly summarized below.

Sayeed Ahmed and Algarni [20] examined the relationship between the temperature change of the disc and the number of modifications in the form of grooves and holes. The heat flux entering the disc did not depend on the temperature in the lining area, which was possible due to the introduction of heat partition coefficient which was constant throughout the braking process. Its value depended on thermal effusivity of the disc and the brake pad material as well as the area of the entire rubbing path on the disc-pad friction surface. Hence, by changing the number of grooves (change in contact surface area), the value of the friction power directed to the disc also changed. The temperature results obtained on the basis of numerical calculations (ANSYS) were experimentally verified using the infrared thermometer.

The analytical model of a multi-disc braking system with the discs made of carbon friction composite material Termar-ADF was proposed in the article by Yevtushenko et al. [21]. The authors calculated temperature of a single brake disc with different length of the fiber bundles of the composite taking into account convection heat transfer with the surrounding air. Based on the developed model with micro, meso, and macro levels, the changes in temperature distributions and their correlations with coefficient of volumetric concentration of bundles as well as the length of the bundles were shown and discussed.

The methodology for selection of materials for disc brake pads was studied using 3D numerical model of the disc brake [22]. Six pads materials were tested in combination with cast iron disc. A number of parameters characterizing the frictional heating process during braking (maximum temperature, braking time, temperature-dependent coefficients of friction and wear rate, braking efficiency, etc.) were accounted for allowing the selection of the optimum combination of materials in the light brake operation mode.

Tribological characteristics (dependence of the coefficient of friction and wear rate on the sliding distance, velocity, and contact pressure) of copper-graphite composites reinforced with Cu-coated or uncoated SiO_2_ were studied in the article [23]. The measurements were carried out on a pin-on-ring tribometer.

A broader review regarding experimental research as well as analytical and numerical modeling of frictional heating in braking systems is presented in the articles [24,25,26].

Comprehensive analysis of braking systems which find application in railway vehicles was presented by Günay et al. [27]. The authors described in detail the method of assessing braking performance of railway vehicles. It is worth noting that an empirical formula, where coefficient of friction is dependent simultaneously on sliding velocity and contact pressure, was given for vehicles equipped with cast iron brake shoes. On the other hand, it is often assumed in numerical braking simulations that the friction coefficient remains constant during braking, regardless of the operating conditions [15,16,17,28]. One of the assumptions made in the present study is that taking into account frictional characteristics dependent on operating conditions may be vital to reliably model frictional heating during braking of railway vehicles equipped with composite brake shoes.

Apart from introduction, the paper is divided into five sections. First, the experimental part of the study is discussed, including description of the full-scale dynamometer, materials used in the research, test program, and results of braking torque measurement. Then, the statement of the boundary value heat conduction problem is given and finite element models of the wheel-brake shoe system are presented. Subsequent section contains results of numerical analysis, namely calculated temperature fields, their discussion and comparison with the experimental measurements. Finally, the study is summarized, and conclusions are drawn.

Experimental tests on inertia test benches (including measurements of temperature, wear, coefficient of friction, etc.), for braking systems of rail vehicles, are usually complicated, labor-intensive, and expensive. Numerical modeling has the potential to be a good alternative for dynamometer tests at the initial stage of the railway braking system design process. Therefore, the main purpose of this article is to evaluate accuracy of temperature fields estimated by numerical calculations concerning long-lasting (order of 103 s) braking process, performed for different friction materials, using 2D and 3D FE models, where variable operating conditions and frictional characteristics are accounted for.

## 2. Experimental Research on a Dynamometer Test Stand for Railway Brakes

**Figure 1:**
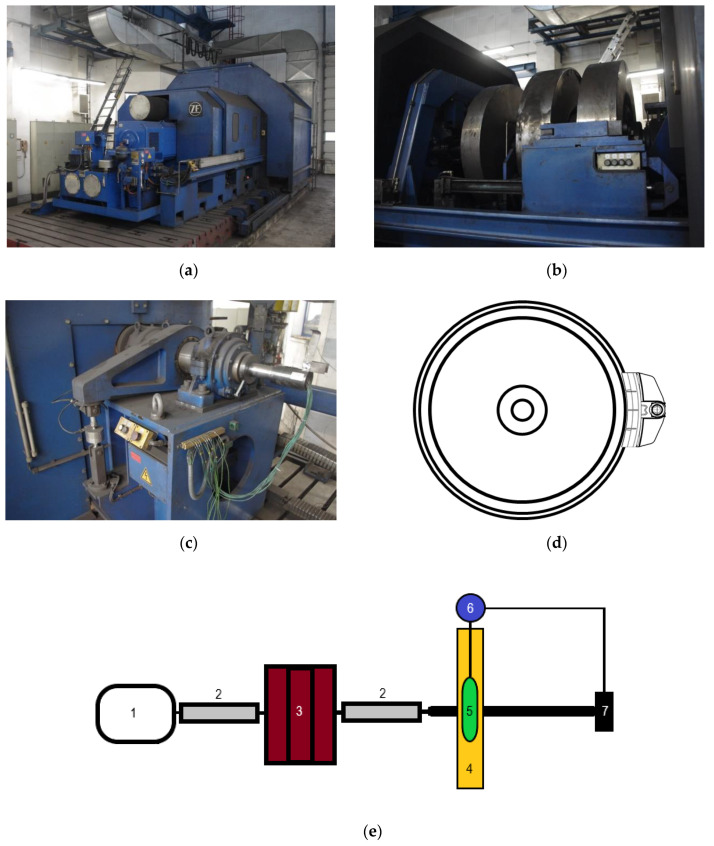
A full-scale inertia dynamometer for testing of brake friction pairs at the Railway Research Institute in Warsaw (**a**–**c**) (pictures courtesy of the Railway Research Institute in Warsaw) and (**d**) 1xBg railway tread brake configuration, (**e**) scheme of the test rig with main components: 1—electric motor; 2—drive shaft; 3—flywheels; 4—railway wheel; 5—brake block; 6—brake cylinder; 7—load cell.

The experimental part of this work was performed on a full-scale inertia dynamometer for testing of brake friction pairs at the Railway Research Institute in Warsaw (Figure 1a–c). The test bench was described extensively in the article by Konowrocki et al. [29]. 

The simplified schematic drawing of the dynamometer is presented in Figure 1e. The drive shafts (2) are coupled to the electric motor (1), flywheels (3), and an axis equipped with railway wheel (4). Flywheels are used to represent the mass of a railway vehicle, the braking of which is simulated during the test (the kinetic energy of the motor-shafts-flywheels-railway wheel system rotational motion corresponds to the kinetic energy of the translational motion of the railway vehicle). If solely combinations of flywheels were used, it would be possible to reach the moment of inertia corresponding only to a few specific railway vehicle masses. To obtain moment of inertia intermediate to the basic values, it is necessary to control the electric motor so as to reduce or supplement the moment of inertia generated by rotating flywheels [30]. Angular velocity of the system is controlled by the rotary encoder (Heidenhain ROD 436, Traunreut, Germany).

The tested friction pair comprised railway wheel (4) and composite brake shoe installed in the holder, part of the brake block (5). Brake block is fixed to the frame which has axis of rotation independent from the motor-shafts-flywheels-railway wheel system but coaxial to it. This way, it is possible to measure friction generated at the wheel-brake shoe interface using load cell (7) (HBM-U2A). The principle of braking torque measurement is presented in Appendix B to UIC Code 548 [30]. Normal force exerted on the brake block comes from compressed-air brake cylinder (6) and was determined using load cell HBM-U2B.

Three K-type thermocouples (NiCr-NiAl, TTE426-K-2 × 0.22-850-1-QQP, Termoaparatura Wrocław, Wrocław, Poland) were installed below wheel running surface to measure wheel temperature in the course of the test. Thermocouples were located on average 5.8 mm below the working surface every 120° along the circumference and at the distance of 110 mm, 85 mm, and 55 mm from the edge of the wheel in the *z*-axis direction, which gives coordinates in the cylindrical system $T_{1E}=$(r=430.62, θ=210°, z=110), $T_{2E}=$ (r=430.24, θ=330°, z=85), $T_{3E}=$(r=430.48, θ=90°, z=55).

**Figure 2:**
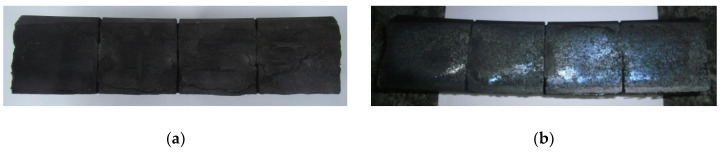
View of the brake shoes made of: (**a**) Material A; (**b**) Material B after tests on a full-scale dynamometer.

Two friction pairs were tested in 1xBg configuration [5,13], i.e., unilateral configuration with a single brake shoe (Figure 1d). ER7 steel wheel and two composite materials were subject of the experimental study, denoted Material A and Material B, respectively. The basic dimensions of the 1xBg brake shoe-wheel system are given in Table 1. The composition of the organic composites under study is given in Table 2, and an exemplary view of the brake shoes after the tests is shown in Figure 2. The materials chosen for the study differ in reinforcing fiber used in the formulation, which has significant impact on their mechanical, thermophysical, and tribological properties. Considerable amount of data acquired in the experimental part of this work allowed verification of the numerical models described in the paper. The results of comprehensive tribological tests of the abovementioned friction pairs, on reduced- and full-scale inertia dynamometer, are presented in the articles [31,32]. Braking time, wheel angular velocity, braking distance, brake cylinder pressure, braking torque, temperature under the wheel running surface, and cooling air flow velocity were measured throughout the test.

The brake shoes were bedded-in before the execution of the main test program by braking at 120 km/h (contact force 30 kN, mass per wheel 7.5 t). The bedding-in procedure was repeated until reaching a minimum of 85% contact area between the brake shoe and the wheel. The main test on a full-scale dynamometer consisted of accelerating the wheel to angular velocity corresponding with the translational velocity of a rail vehicle in real conditions, i.e., equal to 160 km/h, 120 km/h and 80 km/h (for Material A—Figure 2a) and 80 km/h, 120 km/h, and 160 km/h (for Material B—Figure 2b), then braking to a stop. Cooling of the friction pair was performed by rotating the wheel at the angular velocity simulating the translational velocity of 100 km/h. Subsequent braking process commenced when the average temperature measured by three thermocouples below the surface dropped to less than 60 °C. The complete test program is presented in Table 3.

**Figure 3:**
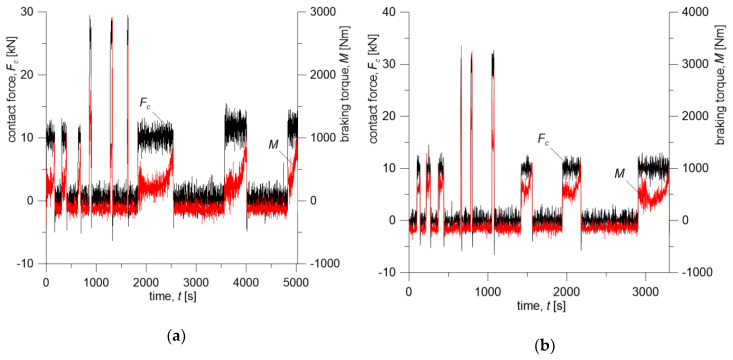
Changes in contact force $F_{c}$ and braking torque M during braking for: (**a**) Material A; (**b**) Material B.

**Figure 4:**
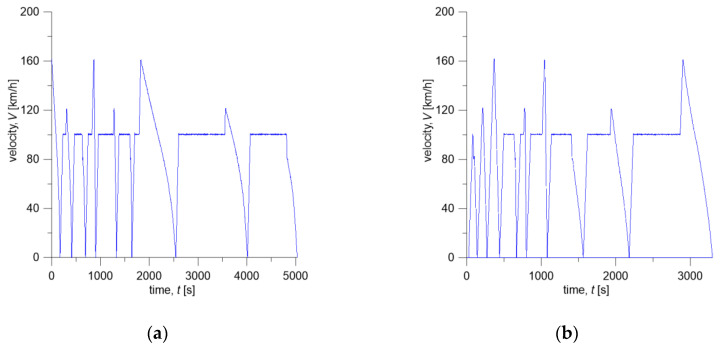
Changes in simulated velocity V of the vehicle during braking for: (**a**) Material A; (**b**) Material B.

Contact force $F_{c}$ time courses and braking torque M measured during tests on a full-scale dynamometer for the considered friction systems are presented in Figure 3. The changes in vehicle velocity during braking are shown in Figure 4.

## 3. Statement of the Problem

At the initial moment of time t=0, wheel of the considered braking system rotates at constant angular velocity $\omega_{0}=V_{0}R_{w}^{-1}$, where $V_{0}$ is the linear velocity on the wheel rolling radius $R_{w}$, equal to the simulated vehicle velocity. Pressed by contact force $F_{c}$, the brake shoe comes into frictional sliding contact with the wheel, which leads to reduction of angular velocity of the wheel until it is stopped. After stopping, the brake is released, and the wheel is accelerated again. The above sequence was repeated in accordance with the test program carried out on the dynamometer (Table 3). The single braking time varied depending on the input parameters (initial velocity, contact force) and material (coefficient of friction, material properties) changing over time.

Assumptions made in the calculations:

1. kinetic energy of rotating masses is entirely converted into heat;
2. deformation of the system components due to mechanical forces and temperature is neglected—only thermal problem is considered;
3. materials of the friction elements are isotropic and their properties depend on temperature;
4. geometrical model of the wheel does not account for holes drilled for thermocouples due to very small diameter of the tip of the thermocouple; such dimension would imply very fine distribution of the finite element mesh in the vicinity of the holes;
5. the outer regions of the brake shoes were not modelled since the temperature in that location does not change during the entire analyzed process;
6. the coefficient of friction changes during braking, and these changes are known *a priori* on the basis of measurements on a full-scale dynamometer;
7. on the free surfaces, convective cooling takes place with the constant heat transfer coefficient, defined separately for specific areas on the surface of the wheel and brake shoes;
8. thermal radiation was not accounted for.

**Figure 5:**
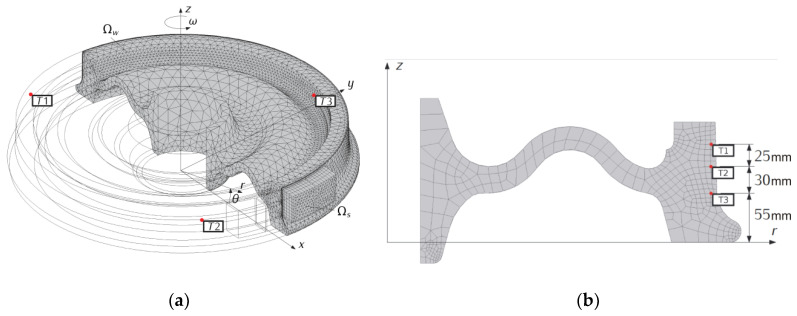
Finite element meshes of the tread brake used in braking simulation: (**a**) spatial; (**b**) two-dimensional (axisymmetric) with measurement points; r—radial coordinate, $T_{1},\;T_{2},\;T_{3}$ —temperature at specific location inside the wheel, θ—circumferential coordinate, $\Omega_{s}$ —region within the volume of the brake shoe, $\Omega_{w}$ —region within the volume of the wheel, ω —angular velocity of the wheel.

Two computational models of frictional heating for 1xBg wheel-brake shoe system were developed and tested: spatial (denoted 3D) and two-dimensional, axisymmetric (denoted 2D)—Figure 5. In the case of the spatial model, changes in the transient temperature field T(r,θ,z,t) of the wheel and brake shoes were determined from the solution of the following nonlinear boundary value heat conduction problem:

(1) $$\frac{1}{r}\frac{\partial }{\partial r}\left(K_{s}\left(T\right)r\frac{\partial T}{\partial r}\right)+\frac{1}{r^{2}}\frac{\partial }{\partial \theta }\left(K_{s}\left(T\right)\frac{\partial T}{\partial \theta }\right)+\frac{\partial }{\partial z}\left(K_{s}\left(T\right)\frac{\partial T}{\partial z}\right)=\rho_{s}c_{s}\left(T\right)\frac{\partial T}{\partial t}\;,\;\;\;0<t\leq t_{s},\;\;\;(r,\theta ,z)\in \Omega_{s},$$

(2) $$\begin{array}{l} \frac{1}{r}\frac{\partial }{\partial r}\left(K_{w}\left(T\right)r\frac{\partial T}{\partial r}\right)+\frac{1}{r^{2}}\frac{\partial }{\partial \theta }\left(K_{w}\left(T\right)\frac{\partial T}{\partial \theta }\right)+\frac{\partial }{\partial z}\left(K_{w}\left(T\right)\frac{\partial T}{\partial z}\right)=\rho_{w}c_{w}\left(T\right)\left[\frac{\partial T}{\partial t}+\omega (t)\frac{\partial T}{\partial \theta }\right]\;,\\ \;\;\;\;\;\;\;\;\;\;\;\;\;\;\;\;\;\;\;\;\;\;\;\;\;\;\;\;\;\;\;\;\;\;\;\;\;\;\;\;\;\;\;\;\;\;\;\;\;\;\;\;\;\;\;\;\;\;\;\;\;\;\;\;\;\;\;\;\;\;\;\;\;\;\;\;\;\;\;\;\;\;\;\;\;\;\;\;\;\;\;\;\;\;\;\;0<t\leq t_{s},\;\;\;\;(r,\theta ,z)\in \Omega_{w}, \end{array}$$

where $K_{s,w}$—thermal conductivity of materials, t—time, $t_{s}$—total braking time, T—temperature, ρ—mass density of materials, c—specific heat capacity of materials. 

In the brake shoe-wheel contact area, denoted Γ, the conditions of perfect thermal contact were assumed:

(3) $$K_{w}\left(T\right){\frac{\partial T}{\partial r}|}_{r=r_{eq}}-K_{s}\left(T\right){\frac{\partial T}{\partial r}|}_{r=r_{eq}}=q\left(r,\;\theta ,\;z,\;t\right),\;\;\;(r,\;\theta ,z)\in \Gamma ,\;\;\;0<t\leq t_{s},$$

(4) $$T_{w}(r,\theta ,z,t)=T_{s}(r,\theta ,z,t),\;\;\;\;(r,\theta ,z)\in \Gamma ,\;\;\;0<t\leq t_{s},$$

where:

(5) $$q(r,\theta ,z,t)=f(t)p(t)V(t),\;\;\;(r,\theta ,z)\in \Gamma ,\;\;\;0<t\leq t_{s},$$

q(r,θ,z,t) is change in time of the total specific power of friction, $p(t)=F_{c}(t)/A_{a}$ is change in time of the contact pressure distributed uniformly on the friction surface $A_{a}$, f(t)—is change in time of friction coefficient, V(t)—is change in time of velocity on the equivalent radius of the wheel.

In the case of a two-dimensional model, friction power generated in the brake shoe-wheel contact area should be averaged using coverage factor $\eta =\theta_{0}/2\pi$. In consequence, calculations for the wheel and brake shoe had to be carried out separately. In addition, taking into account that 3D spatial simulations were performed for both friction elements, only wheel was analyzed in the 2D model. The axisymmetric temperature field T(r,z,t) of the wheel was determined from the following boundary value heat conduction problem:

(6) $$\frac{1}{r}\frac{\partial }{\partial r}\left(K_{w}\left(T\right)r\frac{\partial T}{\partial r}\right)+\frac{\partial }{\partial z}\left(K_{w}\left(T\right)\frac{\partial T}{\partial z}\right)=\rho_{w}c_{w}\left(T\right)\frac{\partial T}{\partial t},\;\;\;0<t\leq t_{s},\;\;\;\;(r,z)\in \Omega_{w}.$$

Part of the friction power density directed towards the wheel was equal to:

(7) $$q_{w}(r,z,t)=\gamma (t)\eta f(t)p(t)\;V(t),\;\;\;(r,z)\in \Gamma ,\;\;\;0<t\leq t_{s},$$

where the heat partition coefficient was calculated using Charron’s formula [33]:

(8) $$\gamma =\frac{\sqrt{K_{w}\rho_{w}c_{w}}}{\sqrt{K_{s}\rho_{s}c_{s}}+\sqrt{K_{w}\rho_{w}c_{w}}}.$$

Initially the wheel and the brake shoes were at ambient temperature $T_{0}=T_{a}$.

**Figure 6:**
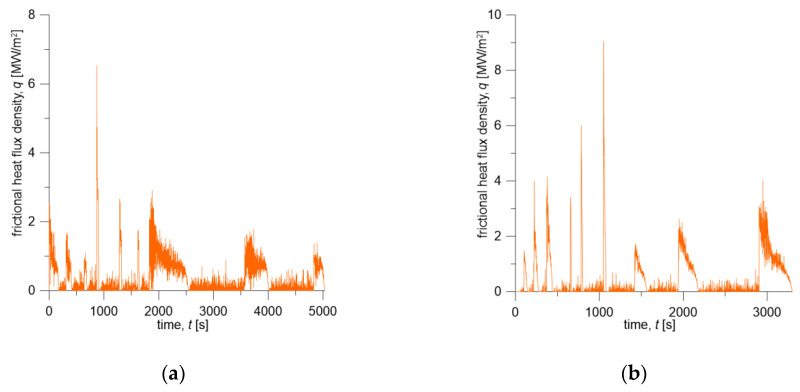
Changes in frictional heat flux density generated in the wheel-brake shoe contact area during braking: (**a**) Material A—ER7; (**b**) Material B—ER7.

Time profile of the friction power density q(t) was calculated from braking torque and translational velocity changes determined on the dynamometer during braking. The product of the coefficient of friction and the contact pressure, appearing in Equation (5), was obtained from the measured braking torque $M(t)=f(t)p(t)A_{a}r_{eq}$. Knowing the velocity change (Figure 4), the friction power density is $q(t)=M(t)V(t)A_{a}^{-1}$ (Figure 6).

## 4. Description of the 3D and 2D Finite Element Models of the Wheel-Brake Shoe System

The purpose of computer simulations of frictional heating was to develop computational models that allow determination of temperature field changes in the volume of the wheel and the brake shoe. Particularly important was the area near the contact surfaces of both objects, including the location of measuring points from the dynamometer tests. Due to the nature of frictional heating, the highest temperature gradients occur in the normal direction from the working surfaces of the contact elements to the outer cylindrical surfaces. In the case of the brake shoe (only 3D model), linear distribution of 8 finite elements in the 1/100 ratio was set in this direction. For the wheel, number of finite elements on the entire circumference of the working surface was increased (relative to the remaining area), with a view to minimizing the total number of finite elements (Figure 5a). Additionally, a thin (of the order of millimeters) surface layer was created.

To ensure accuracy of the results, several finite element meshes were built, starting from coarse grid and increasing the total number of model elements, including elements of the wheel’s surface layer. Due to the complex shape of the wheel and partial contact on the friction surface with the brake shoe, a mesh consisting of tetrahedral, prismatic, and pyramidal elements was used.

When creating the mesh, it was necessary to merge opposite nodes of the brake shoe and wheel surfaces, hence the shape of the elements from the outside of both parts is consistent. These criteria forced a specific approach to dividing the area into spatial elements. The best solution—accuracy-wise—was to use hexagonal elements characterized by regular shapes—the edges are parallel to the direction of the largest temperature gradients. In the analyzed case it was difficult due to the fact that the surface of the brake shoe contacting the wheel is separated by slots. Therefore, the assumed contact area was smaller than determined from nominal width and length of the brake shoe. The first stage in the construction of the mesh was creating of triangular surface elements, then their extrusion from the contact surface to the outer surface of the brake shoe. In this way, prismatic elements were created. In the case of the wheel, a mesh of tetrahedral elements was generated in the entire volume. In the last stage, a layer replacing tetrahedral elements with prismatic ones was created at the working surface. Due to the largest temperature changes in the contact area, variable thickness distribution of the elements in both objects was introduced. The number of boundary layers was equal to 4, the boundary layer stretching factor 1.2, the thickness adjustment factor 1.2, thickness of first layer—automatic.

Preliminary calculations were carried out to verify the approach described above. It was found that further increasing the number of elements and changing their order to a higher one did not affect the temperature distribution. Finally, Lagrange second order finite elements were used to create the model’s mesh (in the case of linear elements temperatures were significantly underestimated). The model comprised 91,794 tetrahedral, 796 pyramid, 19,202 prism, 24,872 triangular, and 1526 quadrilateral elements. The total number of degrees of freedom (DOFs) was 228,138 (plus 67,290 internal DOFs).

By contrast, the two-dimensional model (Figure 5b) was developed using axisymmetric (2D) quadrilateral elements (340 elements). The number of degrees of freedom was 1503.

## 5. Numerical Analysis

Numerical calculations of the space-time temperature distribution in the friction elements of railway tread brake in 1xBg configuration were carried out, using 3D and 2D models described in Section 4, for two combinations of brake shoe-wheel friction pairs (organic composite Material A/ER7 steel and organic composite Material B/ER7 steel).

Initial conditions and operating parameters for the numerical simulations were adopted from the test program carried out on a full-scale dynamometer (Table 3) and are consistent with changes in contact force $F_{c}$ (Figure 3) and braking torque M during repeated braking (Figure 4). Knowing the time courses of $F_{c}$ and M for both friction pairs, changes in friction heat flux densities q (Figure 6) generated on the brake shoe-wheel contact surface (Materials A or B and ER7 steel) were determined.

**Figure 7:**
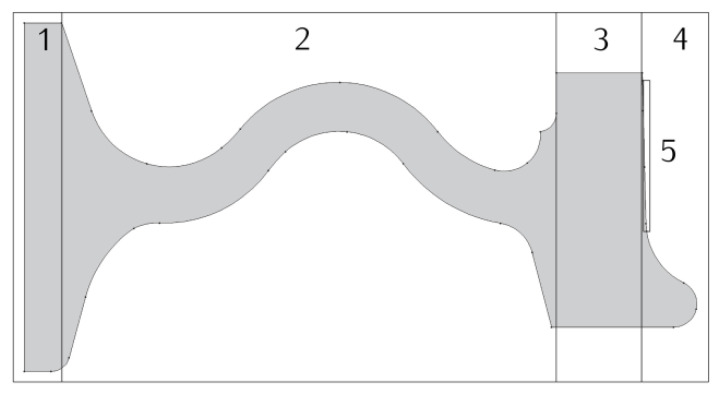
Areas of different convection coefficient h applied in braking simulations [34].

The thermophysical properties of the considered friction pair materials, which were used for calculations using 2D and 3D numerical models, are shown in Table 4 and Table 5. Heat transfer coefficient with the environment h assumes different values in specific areas of the wheel (Figure 7) according to the data listed in Table 6.

As established in Section 2, the temperature measurement inside the steel wheel was made using three K type thermocouples (NiCr-NiAl) located 5.8 mm below the working surface displaced every 120°. The same arrangement of measuring points was reproduced in the 3D spatial model (Figure 5a) and in the simplified 2D axisymmetric model (Figure 5b).

**Figure 8:**
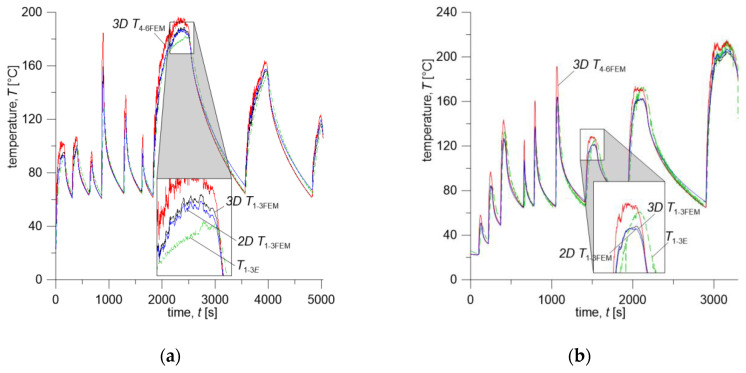
Mean temperature changes: (**a**) Material A; (**b**) Material B; average values from three thermocouples (dashed lines) $T_{1-3E}$, three points from 3D and 2D numerical calculations under the surface of the wheel $T_{1-3FEM}$ and three points on the surface of the wheel 3D $T_{4-6FEM}$.

**Figure 9:**
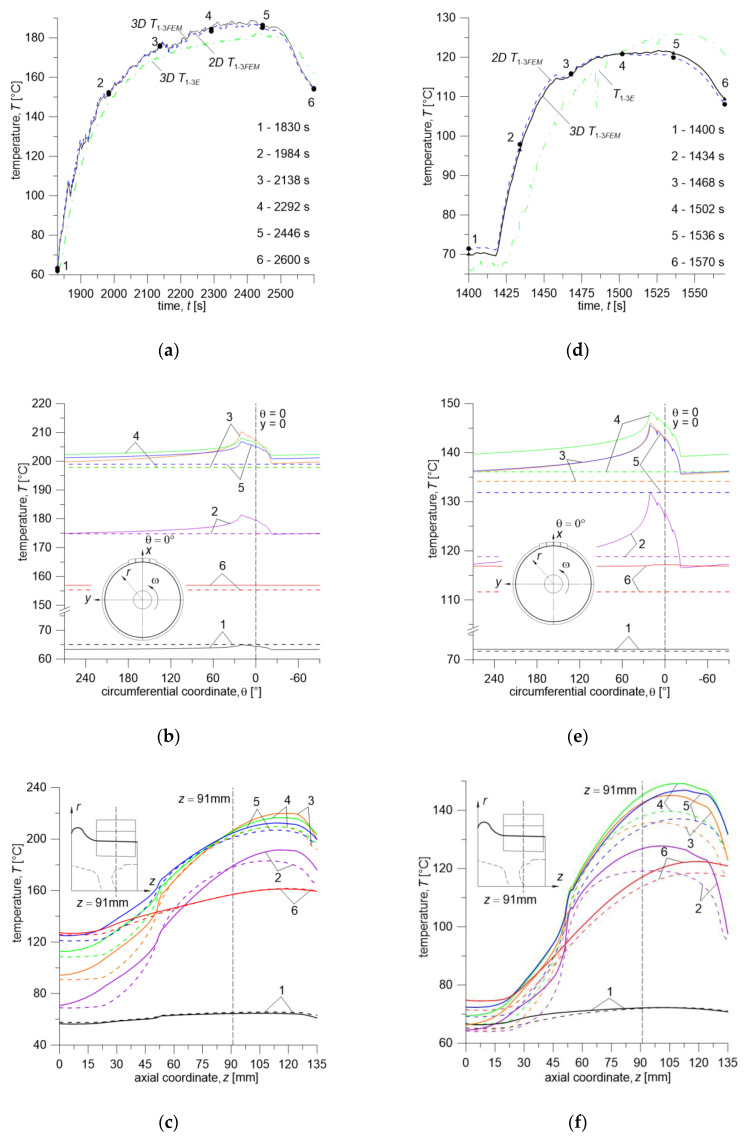
“7-th braking”: (**a**–**c**) Material A/ER7; (**d**–**f**) Material B/ER7.

The curves of experimentally measured and calculated time courses of temperature based on the proposed numerical models are shown in Figure 8 and Figure 9. The temperature values are average values measured by thermocouples ($T_{1-3E}$) and calculated at the corresponding points with the computational models ($T_{1-3FEM}$) at every time step.

Temperature evolutions measured 5.8 mm ($T_{1}=5\;\mathrm{mm}$, $T_{2}=6\;\mathrm{mm}$, $T_{3}=6.5\;\mathrm{mm}$) below the wheel running surface ($T_{1-3E}$) and calculated at respective points with 3D and 2D models ($T_{1-3FEM}$) are presented in Figure 8. Additionally, average temperature on the wheel running surface $T_{4-6FEM}$ was calculated with the 3D model keeping identical distance of the data collection points from the outer face of the wheel rim. The arrangement of points nos. 4–6 located on the wheel running surface is shown on the diagram in Figure 5a.

According to the test program from Table 3, for the friction pair comprising Material A (brake shoe) and ER7 steel (wheel), the first three brake applications (no. 1–3) were carried out successively from initial velocities of 160 km/h, 120 km/h, and 80 km/h, at a contact force of 10 kN and mass per wheel of 2.5 t. During braking no. 1 (160 km/h, 10 kN, 2.5 t), the average temperature measured 5.8 mm below the wheel surface reached maximum value of $T_{1-3E}=92.3\;{}^{\circ}C$, for braking no. 2 (120 km/h, 10 kN, 2.5 t)—$T_{1-3E}=99.2\;{}^{\circ}C$, and for brake application no. 3 (80 km/h, 10 kN, 2.5 t)—$T_{1-3E}=83.2\;{}^{\circ}C$ (Figure 8a). The total time of three braking cycles was $t_{1-3}=866\;s$. It should be noted that braking no. 1 began when the friction elements initial temperature was $T_{0}=23\;{}^{\circ}C$ (cooling air (ambient) temperature was 10 °C), while subsequent brakings until the end of the tests began when the average temperature, measured by three thermocouples 5.8 mm below the wheel running surface, dropped to the value in the range of $T_{1-3E}=62.7÷64.9\;{}^{\circ}C$.

The next stage of the study was the sequence of three brake applications (no. 4–6) initiated, as previously, at velocities of 160 km/h, 120 km/h, and 80 km/h and mass per wheel of 2.5 t, while the contact force was increased to 30 kN (Table 3). Due to the increase in the contact force, the time of each single braking ($t_{4},\;t_{5},\;t_{6}$) shortened, while the total time $t_{4-6}$ including cooling time extended to 966 s. However, due to the more rapid braking process, the maximum temperature values are much higher, especially for brakings no. 4 and 5 at velocities of 160 km/h and 120 km/h—they are equal to $T_{1-3E}=132.2\;{}^{\circ}C$, and 108.7 °C, respectively (Figure 8a).

In the final stage of the test (brakings no. 7–9) mass per wheel was increased to 10 t, and contact force was reduced to 10 kN, while the order of brake applications remained unchanged. Due to the fourfold increase in mass per wheel, the time of the entire sequence significantly lengthened, as compared to the previous two braking cycles 1–3 and 4–6 and is equal to $T_{1-3E}=3686\;s$. Additionally, the maximum values of the measured temperature are much higher and reach values of $T_{1-3E}=182.5\;{}^{\circ}C$, 154.5 °C for individual brake applications (Figure 8a).

The first three braking sequences according to Figure 8a during the time from *t* = 0 to t=3297s were carried out at the contact force $F_{c}=10\;\mathrm{kN}$, which at the nominal area $A_{a}=25250\;{\mathrm{mm}}^{2}$, gave the nominal contact pressure value of 0.396 MPa. The subsequent three brake applications were performed at $F_{c}=30\;\mathrm{kN}$ (pressure of about 1.188 MPa) and the last three again at 10 kN.

The test program presented in this article for the second friction pair, consisting of the brake shoe made of Material B and ER7 steel wheel, differs from the previous one (i.e., Material A/ER7 system) in the order of initial braking velocities, i.e., 80 km/h, 120 km/h, and 160 km/h (Table 3). Considering the thermophysical properties of Material B and Material A (Table 4) and the change in the performed braking sequence, time course of temperature for this friction pair is significantly different (Figure 8b). The difference is pronounced especially in the total braking cycle time, which is equal to t=5032 s for Material A/ER7 pair and t=32 97s for Material B/ER7. This is mainly due to a completely different gradual heating of the wheel, and thus with smaller values of the maximum temperature in the wheel in the initial stage of testing and its faster decrease after subsequent braking to the programmed initial temperature. That difference can also be observed by comparing the time courses of friction power density corresponding to each of the friction pairs, shown in Figure 6.

In the first part of the program consisting of three brake applications no. 1–3, from 80 km/h, 120 km/h, and 160 km/h, with contact force of 10 kN and mass per wheel of 2.5 t, the maximum average temperature measured by three thermocouples located 5.8 mm below the wheel running surface, reached values $T_{1-3E}=49\;{}^{\circ}C$, 83.2 °C, 132.7°, respectively, where its duration was *t*_1–3_ = 652 s. During the subsequent braking sequence (brakings no. 4–6 from 80 km/h, 120 km/h and 160 km/h, at $F_{c}=30\;\mathrm{kN}$ and m=2.5 t) average temperature reached maximum values of $T_{1-3E}=89.5\;{}^{\circ}C$, 120 °C, 158.4 °C, respectively, where *t*_4–6_ = 766 s. Throughout the final part of the program (brake applications no. 7–9 from 80 km/h, 120 km/h, and 160 km/h, at $F_{c}=10\;\mathrm{kN}$ and m=10 t), maximum values of the average temperature are $T_{1-3E}=126\;{}^{\circ}C$, 173 °C, 213.8 °C, respectively, for *t*_7–9_ = 1879 s.

The time courses of temperature calculated with the 2D and 3D FE models show very good agreement with measured values (Figure 8a,b). It can be seen that simplifications assumed in the proposed numerical models, i.e., the condition of equality of temperature on the brake shoe-wheel contact surface, introduction of the heat partition coefficient in 2D system, as well as convective heat transfer coefficient which is constant in time, but different in particular zones of the wheel surface, (see Figure 7), are acceptable and do not significantly affect accuracy of temperature estimation, even for such long and variable brake applications. Both the temperature values calculated from the spatial (3D $T_{1-3FEM}$) and two-dimensional (2D $T_{1-3FEM}$) models slightly increase the temperature compared to the experimental measurement results. The difference between these values is not more than 20% (braking no. 4, Material A and B). Comparing the 3D $T_{1-3FEM}$ and 2D $T_{1-3FEM}$ temperature values calculated at each time step, it can be stated that their difference is negligible (max less than 3%, however, it should be added that for both materials it is higher during cooling and is equal to 7÷9% after braking no. 8). It can be important when choosing the calculation model of frictional heating, especially in terms of the required computing power and “time consumption” concerning model preparation and calculation itself.

In addition, time courses of the average 3D $T_{4-6FEM}$ temperature on the brake shoe-wheel contact surface calculated using the three-dimensional FE model at the condition of temperature equality on that surface were shown in Figure 8a,b.

In the case of friction pairs of the same type as presented 1xBg brake configuration, it must be stated that the initial estimation of the time course of temperature can be carried out using a 2D model, achieving a satisfactory approximation. The temperature calculations were performed on a workstation with following parameters: CPU Intel^®^ Xeon^®^ E5-2698 v4 @ 2.20GHz; RAM 64 GB (DDR4). The calculation times for the Material A/ER7 pair performed were equal to 1h 35 min for the 2D model and 114 h 50 min for the 3D model. However, in the case of the Material B/ER7 friction pair, calculation times were 1 h 13 min and 51 h 32 min, respectively.

In order to perform a detailed analysis of the time courses of temperature (measured—dash dotted lines, and calculated by 2D model—dashed lines and 3D model—solid lines) in the braking system discussed in this work, braking no. 7 for both friction pairs was considered (Figure 9). For Material A/ER7 friction pair, it is braking from 160 km/h at mass per wheel of 10 t and contact force of 10 kN, while for Material B/ER7 pair—80 km/h at mass per wheel of 10 t and contact force of 10 kN. The analysis of single brakings for both cases (plots in Figure 9a,d) confirms the earlier observation that the calculations made using both numerical models approximate the actual time course of temperature very well. Small percentage differences (max 3.37% for Mat. A and 3.3% for Mat. B) between the measured and calculated temperature values may stem both from simplifications imposed on numerical models and measurement errors resulting, among others, from the delay in response of the thermocouples used in experimental part of this study.

The analyzed multiple braking processes are relatively long—they differ significantly from the typical cases of single braking (order of 10^−1^ s) occurring e.g., in motor vehicles. The long duration of the processes, in addition to the large size of the objects such as the wheel of a rail vehicle, with an outer diameter of 870 mm (assumed rolling radius was 437 mm), require a significant computing power and lead to many longer calculations. Therefore, the results obtained in this study, apart from their correctness verified by experimental research, are also intended to justify the previously formulated statement that the 2D axisymmetric model is sufficient to provide results at a similar level of accuracy. In order to perform such analysis, temperature points 1–6 are marked on the time courses of temperature obtained from calculations made with the 2D and 3D models presented in Figure 9a and d. For the abovementioned time points the temperature distributions are shown in Figure 9b and c (Material A/ER7 pair) and Figure 9e and f (Material B/ER7 pair)—for the curve lying on the circumference of the circle in the middle of the brake shoe thickness (Figure 9b,e), and in the axial direction (Figure 9c,f)—for a profile marked in the schematic drawing with a thick line. As can be seen from the time courses of temperature in Figure 9b,e, during braking no. 7 for both considered friction pairs at time points no. 2–5, when the temperature rises and reaches maximum values, this distribution is non-uniform. The maximum temperature fluctuations around the circumference at these points are at a level of 5.5% (Material A) and 13% (Material B), which confirms the assumption that the axisymmetric model will be sufficient to obtain similar results. By contrast, the temperature distribution in the axial direction shown in Figure 9c,f, for both friction pairs, differ by a maximum of 171% for Material A and 115% for Material B. The temperature along the analyzed curve for two friction pairs is higher on the right-hand side of the line *z* = 91 mm due to the lower width of the area absorbing heat through conduction.

**Figure 10:**
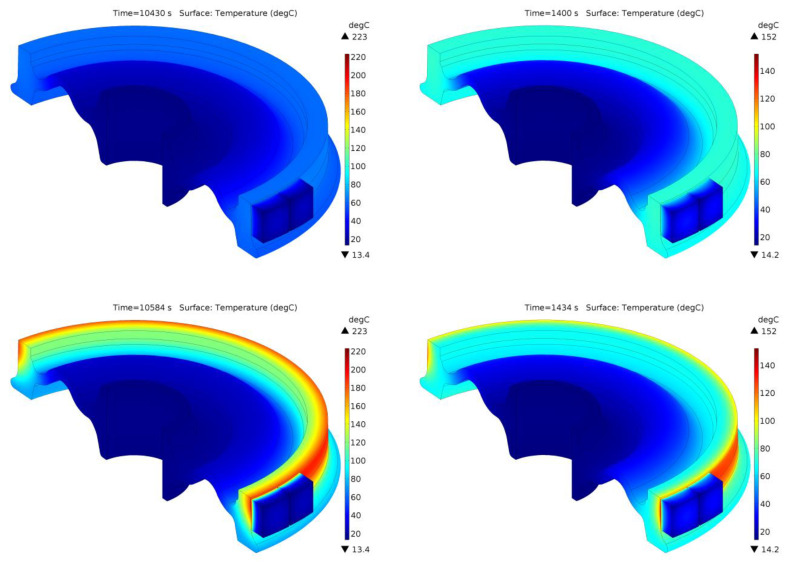

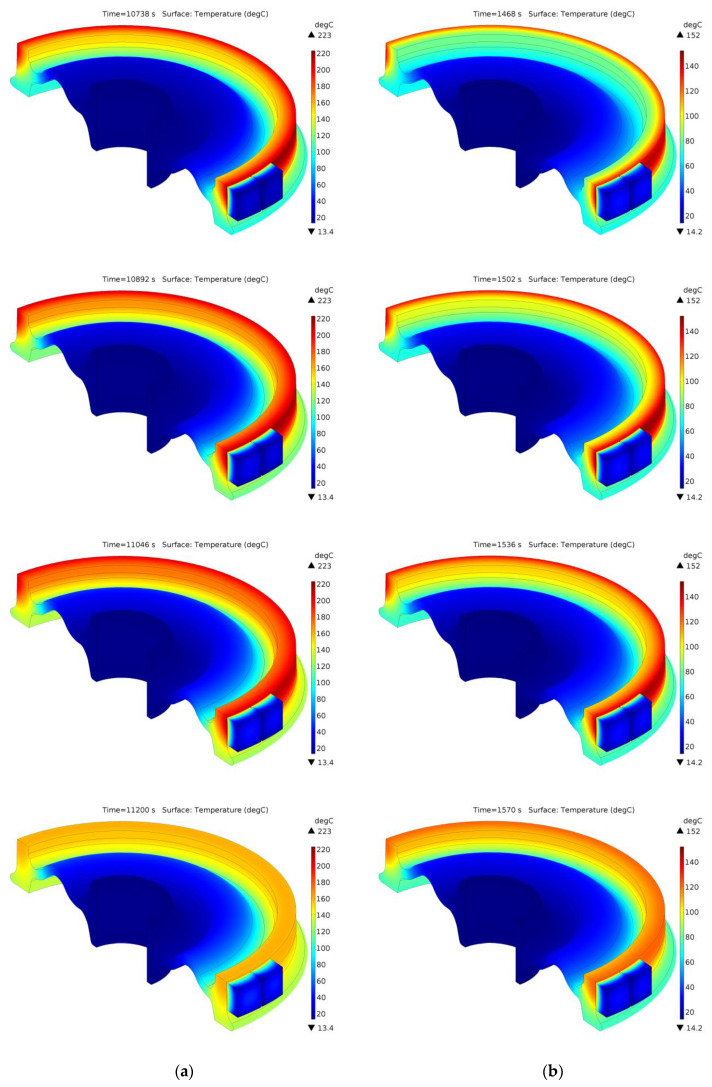
Temperature distribution in the wheel and brake shoe for selected time points marked in Figure 9a,d, for braking no. 7: (**a**) Material A/ER7; (**b**) Material B/ER7.

Temperature distributions inside the brake shoe and wheel during braking no. 7, corresponding to the time points indicated in Figure 9a,d, are presented for the considered friction pairs in Figure 10. In addition to presenting the subsequent stages of temperature change in the wheel and its gradual heating with the braking time, it is also worth noting that the temperature distribution in the wheel at the analyzed time points in both friction pairs is of an axisymmetric nature. The above observation also confirms the possibility of using the 2D FE model to analyze time courses of temperature in 1xBg braking systems during repeated, variable, and prolonged braking.

## 6. Summary and Conclusions

Two 3D and 2D FE numerical models of frictional heating were proposed to calculate temperature fields in the railway tread brake in 1xBg configuration. The consistency of the results obtained for two different friction pairs composed of the brake shoes made of organic composite materials (denoted Materials A and B) and the steel wheel (ER7) demonstrates the usefulness of the developed computational models. 

Temperature values calculated from 3D and 2D models are slightly overestimated as compared to the results of experimental measurements and the difference between these values does not exceed 20%. For this reason, it can be stated that both the 3D and 2D model may be used to successfully estimate the temperature distribution in the friction elements of 1xBg braking system, which is beneficial in the design of new friction pairs in terms of their thermophysical and tribological properties.

Comparison of the temperature calculated using 3D and 2D models at each time step shows that the difference is not greater than 3% for the frictional heating processes and not more than 9% during the cooling steps between successive brake applications. The comparison of models presented in the article proved that 2D frictional heating model can be successfully used to estimate the temperature distributions in the discussed friction node. The computation time (workstation CPU Intel^®^ Xeon^®^ E5-2698 v4 @ 2.20GHz; RAM 64 GB (DDR4)) performed with the 2D model compared to the more general 3D model is reduced by approximately 85 times for the braking cycle lasting 5032 s, and approximately 45 times for the braking cycle lasting 3297 s. 

Both proposed numerical models of frictional heating have certain limitations of applicability. In this article speed and heat flux density determined from experimentally measured braking torque were used as input values. The next stage in the future development of the presented models will be, among others, the mutual coupling of the speed and the friction coefficient through the average temperature on the contact surface of the friction node.

Despite some limitations of the proposed models, they can be used not only to estimate the temperature level during repeated and long-term braking, but also, for example, to study the effect of convective heat exchange on the average wheel temperature or detection of potential wheel overheating related to extreme operating conditions.

The above numerical modeling approach can also be successfully applied to a different type of railway tread brake, e.g., 2xBgu.

The authors of this work did not find any studies where 3D and 2D models of railway tread brake were compared and verified with experimental data, which is also a major asset of this work.

## Figures and Tables

**Table 1:** Dimensions of the braking system.

Parameter	Value
nominal diameter of the wheel $d_{eq}$, m	0.87
nominal surface area $A_{a}$, mm^2^	25,337.91
thickness of the brake shoe, m	0.08
width of the brake shoe, m	0.32

**Table 2:** Formulation of the organic composites, wt% [31,32].

	Base Formulation	Glass Fibre	Steel Fibre
Material A	65–75%	25–35%	0%
Material B		0%	25–35%

**Table 3:** Full-scale dynamometer test program for 1xBg configuration.

Number of Braking	$\mathrm{Initial}\;\mathrm{Velocity}\;\mathrm{of}\;\mathrm{the}\;\mathrm{Vehicle}\;V_{0},\;\mathrm{km}/h$	$\mathrm{Contact}\;\mathrm{Force}\;F_{c},\;\mathrm{kN}$	$\mathrm{Initial}\;\mathrm{Temperature}\;T_{a},\;{}^{\circ}C$	Mass per Wheel *m*, t	Comments
1	160		ambient	2.5	
2	120	10	50–60		
3	80				
4	160				
5	120	30			Material A/ER7
6	80				
7	160				
8	120	10		10	
9	80				
1	80		ambient	2.5	
2	120	10	50–60		
3	160				
4	80				
5	120	30			Material B/ER7
6	160				
7	80				
8	120	10		10	
9	160				

**Table 4:** Properties of the brake shoe materials [31].

		Specific Heat Capacity $c_{s}$, J/(kg K)		Thermal Conductivity *K_s_*, W/(m K)		Mass Density $\rho_{s}$$,\;\mathrm{kg}/\;m^{3}$	Thermal Diffusivity $k_{s}$$,\;m^{2}/s$
at temperature		30 °C	100 °C	30 °C	100 °C	20 °C	
brake shoe	material A	870	1040	1.18	1.47	1930	7.013·10^−7^
	material B	730	860	1.41	1.74	2350	8.594·10^−7^

**Table 5:** Thermal properties of the wheel material (ER7 ^1^ steel) [34].

Temperature, °C	Thermal Conductivity $K_{w}$, W/(m K)	Specific Heat Capacity $c_{w}$, J/(kg K)
0	47.3	440
20	44.1	510
400	39.3	570

^1^ Density of the wheel $\rho_{w}=7850\;\mathrm{kg}\;m^{-3}.$

**Table 6:** Convection coefficient values in the various wheel surface zones [34].

Zone	Heat Transfer Coefficient $h,\;{W/(m}^{2}\;K)$
1	32.6
2	55.9
3	55.9
4, 5	65.3

## Nomenclature

$A_{a}$	nominal area of the contact region between brake shoe and wheel (m^2^)
c	specific heat capacity (J/(kg K))
$d_{eq}$	nominal wheel outer diameter $d_{eq}=2r_{eq}$ (m)
f	coefficient of friction (dimensionless)
$F_{c}$	contact force (N)
h	heat transfer coefficient (W/(m^2^ K))
k	thermal diffusivity (m^2^/s)
K	thermal conductivity (W/(m K))
m	braking mass per one wheel (kg)
M	braking torque (N m)
p	contact pressure (MPa)
q	specific friction power (W/m^2^)
r	radial coordinate (m)
$r_{eq}$	equivalent radius of the contact region (m)
$R_{w}$	equivalent radius of the wheel $R_{w}=r_{eq}$ (m)
t	time (s)
$t_{s}$	total braking time (s)
T	temperature (°C)
$T_{a}$	ambient temperature (°C)
$T_{0}$	initial temperature (°C)
$T_{1-3}$	average temperature from $T_{1}$, $T_{2}$ and $T_{3}$ (°C)
$T_{4-6}$	average temperature from $T_{4}$, $T_{5}$ and $T_{6}$ (°C)
$T_{1},\;T_{2},\;T_{3}$	temperature at specific location inside the wheel (°C)
$T_{4},\;T_{5},\;T_{6}$	temperature at specific location on the contact surface of the wheel (°C)
V	velocity on the equivalent radius of the wheel (vehicle velocity) (m/s)
$V_{0}$	initial vehicle velocity (m/s)
z	axial coordinate (m)
Greek symbols	
γ	heat partition coefficient (dimensionless)
Γ	contact region of the brake shoe and wheel (m^2^)
η	coverage factor (dimensionless)
θ	circumferential coordinate (rad)
ρ	mass density (kg/m^3^)
ω	angular velocity of the wheel (rad/s)
$\omega_{0}$	initial angular velocity of the wheel (rad/s)
$\Omega_{s}$	region within the volume of the brake shoe (m^3^)
$\Omega_{w}$	region within the volume of the wheel (m^3^)
Subscripts	
s, w	brake shoe, wheel
E	experiment
